# Cancer treatment–related cognitive impairment

**DOI:** 10.1093/noajnl/vdag064

**Published:** 2026-03-19

**Authors:** Bryan J Neth, Sanne B Schagen, Jeffrey S Wefel

**Affiliations:** Department of Neurology and Medical Oncology, Mayo Clinic, Rochester, Minnesota, USA; Department of Psychosocial Research and Epidemiology, Netherlands Cancer Institute, Amsterdam, The Netherlands; Department of Psychology, University of Amsterdam, Amsterdam, The Netherlands; Section of Neuropsychology, Department of Neuro-Oncology, The University of Texas MD Anderson Cancer Center, Houston, Texas, USA; Department of Radiation Oncology, The University of Texas MD Anderson Cancer Center, Houston, Texas, USA

**Keywords:** cancer, cognition, cognitive function, neurotoxicity

## Abstract

There is a growing number of cancer survivors given improvement in early cancer diagnosis, therapeutics, and supportive care strategies. Cognitive impairment is among the most common and clinically significant symptoms managed by cancer survivors, which negatively impacts quality of life, functional and occupational status for patients and their care partners. Cancer-related cognitive impairment is often multifactorial—impacted by patient and cancer-specific factors. For systemic (non-CNS) malignancies, cognitive impairment has been identified in up to 75%, depending on cohort, which persists in up to 35% of patients in the years following initial treatment. The most common areas of impairment include memory and processing speed, and executive function. These adverse effects of cancer treatment have been reported after chemotherapy, hormone therapy, targeted therapy, and immunotherapy. This review will summarize existing knowledge regarding the nature and pattern of cognitive changes, mechanisms and risk factors underlying these changes, and biomarkers to support identification of this adverse consequence of cancer therapies.

Key Points

- Cognitive impairment is among the most common and clinically significant symptoms managed by cancer survivors, which negatively impacts quality of life, functional and occupational status for patients and their care partners.
- Cognitive impairment has been identified in up to 75% and persists in up to 35% of patients in the years following initial treatment.
- Ongoing efforts are needed to clarify mechanisms and risk factors underlying these changes, and determine biomarkers to support identification of this adverse consequence of cancer therapies.

Cancer is common, with an estimated 2 million new diagnoses in the United States and 20 million worldwide each year.^1^ There is a growing number of cancer survivors given improvement in early cancer diagnosis, therapeutics, and supportive care strategies, estimated around 18 million people in the United States.^1^ As the population of cancer survivors has grown, there has been an increased emphasis on understanding long-term effects of cancer and its treatment.^2^ Cognitive impairment is among the most common and clinically significant symptoms managed by cancer survivors, which negatively impacts quality of life, functional and occupational status for patients and their care partners.^3–8^ Longitudinal studies of systemic cancer survivors have shown that measurable cognitive impairment is present in up to 30% of patients even prior to starting treatment, this increases up to 75% shortly after treatment, and persists or progresses in the years following treatment in up to 35%.^5^^, ^^8^^, ^^9^ Importantly, impaired cognitive function has been associated with both decreased adherence to cancer therapies^10^ and worse survival in cancer patients.^11–14^

Cancer-related cognitive impairment (CRCI) is often multifactorial—impacted by patient and cancer-specific factors.^4^ Among the most well-known contributors to CRCI is cancer treatment. The association between increased cognitive dysfunction and cancer treatment has been known for decades—with much of the literature focused on “chemobrain” or “chemo fog” in patients with breast cancer.^15^ However, CRCI and treatment-related cognitive impairment occur in patients with many cancer types, including prostate, lung, hematologic malignancies, colorectal cancer, brain cancer, among others.^3–5^ Recent research has focused on better understanding the pathophysiologic mechanisms underlying cognitive impairment related to cancer treatments. While these insights may not offer immediate solutions, they will inform future strategies to better prevent or manage cognitive side effects.

The overall focus of the current review will be on cancer treatment–related cognitive impairment (CTRCI) in adults who have systemic malignancy (without cerebral metastasis) or central nervous system (CNS or brain) cancers.^16–18^ First, we will review the broader context of CRCI, addressing the epidemiology and clinical presentation, providing background into early and late effects of CRCI, as well as risk factors for the development of CRCI. We will then focus on the effects of antineoplastic treatments on cognitive outcomes in cancer patients (CTRCI) and review the contribution of key treatment modalities (eg traditional chemotherapy, hormone therapy, targeted/molecular therapies) for CNS and non-CNS cancers and provide background into pathophysiologic mechanisms contributing to CTRCI. Lastly, we will close with an overview of challenges and promising future directions that will guide the development of preventative and therapeutic strategies. Note that the current review focuses on the contribution of systemic therapy to CRCI. Other important treatment-related contributors to CRCI, including the effect of immunotherapy and radiation therapy, will be reviewed elsewhere.

## Epidemiology and Clinical Presentation

### Incidence

Cognitive impairment is increasingly recognized as a common and distressing sequelae of cancer treatment.^2^^, ^^4^ This is important given improvements in longevity and a greater number of cancer survivors.^2^ The reported incidence and prevalence vary widely across studies, which is due to differences in patient populations, cancer type, specific treatment, timing of cognitive assessment, and cognitive measurement techniques.^4^^, ^^8^ For systemic (non-CNS) malignancies, cognitive impairment has been identified in up to 75%, depending on the cohort, which persists in up to 35% of patients in the years following initial treatment.^5^^, ^^8^^, ^^9^ Given the nature of brain cancers, particularly infiltrating tumors (eg glioma), cognitive impairment has been identified in up to 90% of patients and often persists and worsens throughout the course of cancer.^19^ The clinical presentation of cognitive impairment differs by cancer type, with the key distinction between CNS versus non-CNS involvement, but often includes multiple cognitive domains.^20–22^

### Clinical Features and Symptomatology

**Figure 1.**
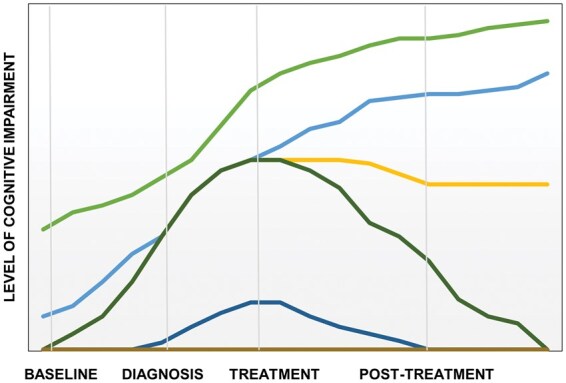
Hypothetical representation of individual cognitive trajectories in cancer survivors.

The clinical features and presentation of CRCI and cognitive effects from cancer treatment (CTRCI) vary widely (Figure 1) depending on multiple factors, including: baseline level of cognitive impairment or symptoms, risk factors (eg age, APOE genotype), comorbid conditions, cancer type/stage and treatment exposures.^3–5^^, ^^8^^, ^^23–25^ Patients may experience subjective (self-reported) cognitive concerns and/or objective (cognitive testing) cognitive impairment from the time of initial diagnosis, treatment, and long after initial treatment.^4^^, ^^16^^, ^^20^ Both subjective cognitive complaints and objective impairment affect quality of life and functional status.^5^^, ^^9^^, ^^26–29^ However, they often do not align, which is not unique to oncology.^3^^, ^^4^^, ^^16^^, ^^20^^, ^^30^^, ^^31^ Self-reports tend to reflect emotional and psychosocial factors more than neuropsychological tests do. In clinical care, both perspectives are considered. In research, however, the choice of measurement method should depend on the research question. For example, if the goal is to determine how treatment affects brain and cognitive function, it is crucial to include neuropsychological assessments.

The specific cognitive domains affected in cancer survivors and after cancer treatments can vary widely as a function of a variety factors, including treatment type, intensity, comorbidities, and timing. The most common areas of impairment include memory and processing speed, and executive function.^4^^, ^^8^^, ^^16^^, ^^25^^, ^^32^ This varies widely by study and patient group. There is no unified clinical course that can explain CRCI. Some patients will not experience any cognitive changes, some patients will have cognitive impairment at time of diagnosis and be relatively unchanged throughout management course, some patients will have increased dysfunction throughout treatment, which can improve (short interval or delayed), and some patients will have progressive or persistent cognitive dysfunction in the years following cancer diagnosis and initial treatment (Figure 1).^4^^, ^^5^^, ^^8^^, ^^16^^, ^^25^ Of course, at times of cancer recurrence/relapses, cognitive function can also be affected, particularly if other treatments are administered at that time. Please refer the previous comprehensive reviews describing the clinical symptomatology and cognitive trajectories in cancer survivors.^4^^, ^^5^^, ^^8^^, ^^16^^, ^^25^^, ^^33^^, ^^34^

## Assessment and Diagnosis of CRCI

The National Comprehensive Cancer Network (NCCN) Clinical Practice Guidelines in Oncology—Survivorship: Cognitive Function Version 2.2025 provides a reasonable framework for oncologists and other clinicians to utilize when evaluating patients with cognitive concerns.^35^ After ruling out focal deficits warranting neuroimaging and potentially reversible contributors (eg vitamin deficiencies, endocrinopathies), providers are encouraged to refer patients for neuropsychological evaluation. Formal neuropsychological assessment, including tests of cognitive function as well as self-report measures of cognitive concerns, mood, fatigue, and other symptoms are important to obtain as part of any assessment of cognitive outcomes. Identifying the nature, extent, impacts, and clinical correlates of cognitive concerns and impairment is clinically relevant to optimally assist patients in maximizing their quality of life.^3–5^^, ^^8^^, ^^16^^, ^^20^^, ^^25^ Neuropsychological research varies by institution and researchers preference, which in the past has made it challenging to compare study results. Consensus recommendations from the International Cognition and Cancer Task Force (ICCTF) (Table 1) are that at least 3 standard tests (that accounts for the most common effected domains) should be included in future studies. These tests include the Hopkins Verbal Learning Test-Revised (HVLT-R; episodic verbal learning and memory), Multilingual Aphasia Examination Controlled Oral Word Association (COWA; verbal fluency and executive function), and Trail Making Test (TMT; psychomotor speed, attention, and executive function).^36^ There are numerous self-report measures with items asking about cognitive concerns; however, several questionnaires are most used in the cancer patient population, including Functional Assessment of Cancer Therapy-Cognitive Function (FACT-Cog), European Organization for the Research and Treatment of Cancer Quality of Life Questionnaire (EORTC QLQ-C30), and the MD Anderson Symptom Inventory (MDASI).^3–6^^, ^^20^^, ^^25^^, ^^36^^, ^^37^

**Table 1.** Tests and measures to consider in a neuropsychological assessment of CTRCI

Domain	Test/measure
Episodic learning and memory	Hopkins Verbal Learning Test—Revised^a^
Processing speed and executive function	Trail Making Test^a^
Expressive language—verbal fluency	MAE Controlled Oral Word Association^a^
Cognitive concerns	FACT—Cognitive Function PROMIS Cognitive Concerns PROMIS Cognitive Abilities
Mood	Hospital Anxiety and Depression Scale Beck Depression Inventory—II Beck Anxiety Inventory
Symptoms	MD Anderson Symptom Inventory^b^
Health related quality of life	FACT—General^b^ (+/-disease specific module) EORTC—Quality of Life Questionnaire (C30)^b^

a Recommended by the ICCTF as a core measurement set.

b The general measure with or without a disease-specific module.

Abbreviations: EORTC, European Organization for the Research and Treatment of Cancer; FACT, Functional Assessment of Cancer Therapy; MAE, Multilingual Aphasia Examination.

## Risk Factors

Several factors increase the risk of the development of CRCI.^5^^, ^^8^^, ^^16^ Established risk factors for the development of age-related cognitive decline (eg Alzheimer’s disease) are the most well-established factors contributing to risk for impairment in cancer survivors, particularly advanced age.^3^^, ^^4^^, ^^8^^, ^^25^^, ^^38^ Older age is associated with increased risk for cognitive impairment, which may predispose to treatment-related effects, including chemotherapy.^39–44^ Baseline patient comorbidities, including the presence of neurologic and psychiatric diagnosis has also been shown to increase the risk of neurotoxicity and cognitive decline after cancer treatment. General medical health comorbidities, including the presence of cardio/cerebrovascular disease, diabetes mellitus, renal disease, as well as mood-related disorders, including depression or anxiety, may contribute to increased likelihood for development of cognitive impairment.^3^^, ^^43^^, ^^45–47^ Baseline cognitive reserve variables (eg education) and baseline cognitive performance also have been associated with longitudinal cognitive outcomes in cancer survivors.^40^

**Figure 2.**
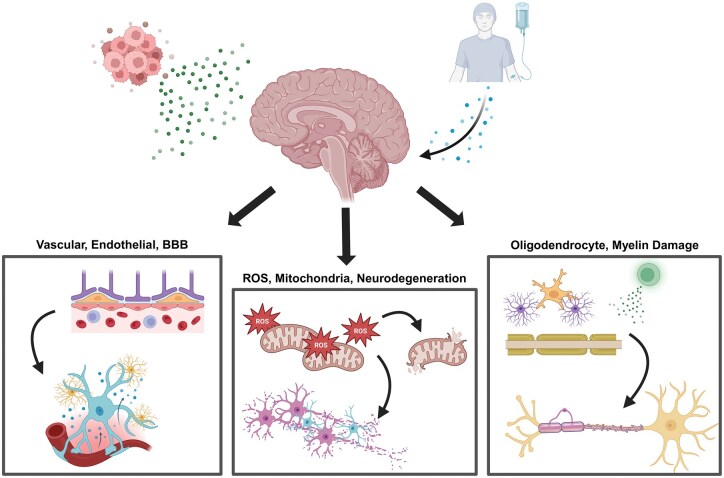
Factors contributing to CRCI. Cancer–related cognitive impairment is influenced by multiple factors, including patient, cancer, and cancer treatment–related factors.

Genetic factors also influence the risk for CRCI and treatment-related cognitive decline.^3^^, ^^8^^, ^^25^ Apolipoprotein epsilon 4 (APOE4) is an established risk factor for age-related cognitive decline due to Alzheimer’s disease and other age-related disorders.^48^^, ^^49^ In the early 2000s, Ahles et al. showed that APOE4 was a risk factor for the development of chemotherapy-related cognitive impairment in long survivors of breast cancer and lymphoma.^50^ This finding has been replicated, and multiple studies have shown that the carriage of the APOE4 allele increases risk for cognitive impairment in patients with cancer.^39^^, ^^51–53^ The carriage of APOE 4 has also been shown to be related to cognitive impairment after radiation for brain metastasis.^54^ Genetic polymorphisms of catechol-O-methyltransferase (COMT, which affects dopamine) and brain-derived neurotrophic factor (BDNF, which is involved in neurogenesis and synaptic plasticity) have also been related to cognitive outcomes in cancer survivors.^55–58^  Figure 2 highlights the patient, cancer, and cancer treatment–related factors that contribute to CRCI.

## Cancer Treatment–Related Cognitive Impairment

With the more widespread use of chemotherapy/antineoplastic therapy, reports of neurotoxicity were published in the 1970s.^59^ Much of the early literature regarding cognitive toxicity was variable and often used broad descriptions describing clinical symptoms and features, including for neuropsychiatric complications.^60^^, ^^61^ With further awareness of cognitive dysfunction related to cancer treatment, there has been improved characterization of specific cognitive domains and clinical phenotypes. Early studies in the 1990s and early 2000s formed the basis for CTRCI and the importance of assessing cognitive outcomes in patients with cancer.^20^ Since this time, there have been numerous cross-sectional studies and increasingly more longitudinal studies that have associated specific antineoplastic agents with cognitive impairment.^62^^, ^^63^ However, there is variability in the effect size of individual therapies on specific cognitive domains. Additionally, meta-analyses also have shown inconsistent findings.^23^^, ^^64–71^ Data are limited by variability in cognitive tests utilized across studies, patient population, and duration of monitoring.^20^ An important consideration is not only the specific therapy but also the dose (for a given patient), and the duration of treatment. Longer exposure to therapy has been shown to increase the risk for the development of CTRCI.^65^ Despite some disagreement, there are several common systemic cancer therapies that have been shown to impact cognitive outcomes, which we will review below.

### Chemotherapy

Traditional cytotoxic chemotherapies are a cornerstone of cancer treatment and target rapidly dividing cells by interfering with proliferation and tumor cell growth. Broad categories include alkylating agents, antimetabolites, anthracyclines, microtubule inhibitors. These therapies are nonspecific and thus are associated with multiple adverse effects such as nausea, diarrhea/constipation, fatigue, bone marrow suppression, cardiotoxicity, and neurotoxicity (eg peripheral neuropathy, cognitive impairment).^72^^, ^^73^

#### Antimetabolites

Antimetabolites impact the ability to produce DNA, due to being structural analogs of purine/pyrimidines bases or by inhibiting enzymes necessary for production of nucleotides.^74^^, ^^75^. Methotrexate is used primarily for management of hematologic malignancies (eg ALL, non-Hodgkin lymphoma, CNS lymphoma) and is among the most well-recognized chemotherapies related to cognitive impairment, especially with high dose regimen.^76^^, ^^77^ Methotrexate has been associated with deficits, including impairment in memory, attention, executive function, and processing speed.^3^^, ^^77–82^ Cerebral white matter changes, leukoencephalopathy, are a well-established finding that can be seen after methotrexate exposure, correlating with the extent of clinical symptoms.^79^^, ^^81^^, ^^83^ Methotrexate has been the focus of extensive basic studies to determine the mechanism of neurotoxicity. Mechanisms leading to cognitive impairment include microglial-dependent reduction in BDNF expression that ultimately leads to loss of activity-regulated myelination,^84^^, ^^85^ and a permanent reduction in oligodendrocytes and their progenitors.^86^ Additionally, methotrexate has been shown to suppress hippocampal cell proliferation impacting cognition.^87–89^ 5-FU, which is used primarily for colorectal cancer and other gastrointestinal malignancies, has been shown to lead to cognitive impairment, including deficits in memory, attention, executive function, and verbal fluency.^90^^, ^^91^ Similar to methotrexate, 5-FU also reduces long-term survival and cell proliferation within the hippocampus and reduces oligodendrocyte precursor cell populations.^92–94^ Cytarabine (Ara-C) is an antimetabolite often used for hematologic malignancies (eg AML, lymphoma) and leptomeningeal disease and has long been associated with the acute neurotoxic syndrome, particularly, with high-dose cytarabine.^95^ Clinical findings include symptoms typical of cerebellar dysfunction and cognitive changes such as generalized encephalopathy, somnolence, confusion, disorientation, and memory loss.^95^ Symptoms are often reversible, although severe cases can have lasting motor and cognitive impairments. This is thought to be related to direct neurotoxicity as well as oxidative stress induced by metabolites.^95^

#### Anthracyclines

Anthracyclines such as doxorubicin and daunorubicin disrupt DNA synthesis by affecting the function of an important enzyme for DNA replication (topoisomerase).^96^^, ^^97^ Doxorubicin is used for the treatment of multiple cancers, including hematologic malignancies, breast, and ovarian cancer, among others. Doxorubicin, which can be used in combination regimens, has specifically been associated with persistent cognitive deficits in memory and executive function, particularly, in patients with breast cancer.^98–100^ There are several proposed mechanisms that may contribute to cognitive toxicity, including the induction of oxidative stress and inflammatory pathways, negatively impacting synaptic plasticity, and reducing neurotransmitter levels including acetylcholine.^98^

#### Taxanes

Taxanes impact microtubule structure, thereby disrupting mitosis and cell division.^101^^, ^^102^ Paclitaxel and docetaxel are commonly used taxanes with well-established neurotoxicity, including peripheral neuropathy.^103^ However, paclitaxel has also been associated with central neurotoxicity, including cognitive impairment with an impact on attention and executive function.^104^^, ^^105^ Given limited CNS penetration, proposed mechanisms include increased pro-inflammatory cytokines and resultant neuro-inflammation, which may lead to a negative downstream impact on neurogenesis.^103^

#### Alkylating Agents

Alkylating agents impede cell replication by cross-linking DNA.^106^ Cyclophosphamide is used for multiple cancers, including lymphomas and breast cancer and has been associated with impairment in multiple cognitive domains.^107–109^ It is important to note that cyclophosphamide is often utilized in combination treatment regimens that confounds cognitive outcomes data. Potential mechanisms include increased oxidative stress, neuroinflammation, inducing apoptosis of neuronal cell populations and impact on hippocampus-dependent learning and memory.^110^ Cyclophosphamide is also used for other indications, including autoimmune disorders, and while cyclophosphamide may be associated with cognitive impairment in patients with cancer, it has been shown to provide benefit for cognition in other patient populations, such as multiple sclerosis.^111^ Other alkylating agents, including nitrosoureas, ifosfamide, and busulfan, have also been associated with general encephalopathy and cognitive impairment, with similar mechanisms including oxidative stress, neuroinflammation, direct DNA damage, potential disruption of hormone production.^112–115^ Temozolomide is the most common alkylating agent utilized in Neuro-Oncology for the management of glioblastoma and other glioma.^116^^, ^^117^ While temozolomide has been shown to impact cognitive performance in animal models,^118–120^ clinical data show varying results, which is impacted by brain tumor infiltration and often the need for radiation therapy. The results of the EORTC 22033-262033 study showed no difference in health-related quality of life or MMSE performance at 1 year in patients with low-grade glioma treated with RT versus temozolomide.^121^ In the CeTeG/NOA-09 trial of temozolomide versus temozolomide/CCNU in newly diagnosed MGMT promoter methylated glioblastoma, patients who received temozolomide alone had higher mini-mental status examination scores at a median of 15-month follow-up, although specific tests of cognitive function and health-related quality of life were not different between groups.^122^ RTOG 0424 monitored cognitive function of patients with high-risk low-grade glioma over the first year of concurrent chemoradiotherapy utilizing dedicated neuropsychological tests. RCI-determined cognitive decline occurred in 50% and 40% at 6 and 12 months, respectively, while health-related quality of life scores were stable over time.^123^

#### Platinum-Based Therapy

Cisplatin, carboplatin, and oxaliplatin are platinum-based antineoplastic agents used to treat multiple solid tumors including lung, testicular, ovarian, as well as can be used as a treatment option for recurrent high-grade glioma, which work by cross linking with purine bases that damage DNA and interfere with DNA repair mechanisms.^124^ Common side effects include neurotoxicity (peripheral neuropathy), ototoxicity, nephrotoxicity, and nausea/emesis.^125^ Cisplatin specifically has been commonly associated with slowed cognitive processing, difficulty with concentration and memory.^126^ While there remains debate with regards to cognitive deficits related to platinum-based therapy, multiple studies have shown clear cognitive impairment after use of cisplatin, particularly in long-term survivors.^127^^,^^128^

### Hormone/Endocrine Therapy

Hormone or endocrine therapy is commonly used in treatment for breast, ovarian, and prostate cancer, with a goal of preventing hormone-stimulated cancer growth. Mechanisms of action include decreased hormone production, or by specifically blocking of hormone signaling, which leads to improved survival outcomes.^129–131^ Hormone therapy has been associated with cognitive impairment, particularly in patients with breast and prostate cancer.^66^^, ^^132^

#### Breast Cancer

Approximately 75% of breast cancer cases are hormone receptor-positive (HR+). To reduce recurrence risk, patients with primary HR+ breast cancer are typically offered (neo)adjuvant systemic therapy, most commonly endocrine therapy (ET). Standard ET duration is 5 years, extendable to seven to ten years for high-risk tumors. In metastatic disease, ET may also be prolonged due to the increasing efficacy of targeted therapy combinations. Tamoxifen is the first-line treatment for premenopausal patients, usually prescribed for at least 5 years. Postmenopausal patients often receive tamoxifen for 2 to 3 years, followed by an aromatase inhibitor.^133^^, ^^134^ Note that many studies assessment effects of hormone therapy include variable cognitive assessment, duration of assessment, and often include heterogenous cohort of patients who also receive chemotherapy which can impact interpretability of results.

#### Estrogen Receptor Modulators and Degraders

Selective estrogen receptor modulators (SERM) block estrogen binding to the estrogen receptor.^133^ Tamoxifen is the most commonly used SERM that exerts tissue-specific agonist or antagonist effects. In the brain, the distribution of ERα and ERβ varies by region, and these receptors are key mediators of estrogen’s influence on cognitive processes. Since tamoxifen can cross the blood-brain barrier and bind to ERs, it may disrupt normal estrogen signaling and thereby impact cognitive function. Tamoxifen has been associated, although not consistently, with lower scores in verbal learning and memory, executive functioning, and processing speed,^135–138^ which may improve upon cessation of therapy.^139^^, ^^140^ A recent study showed that the cognitive effects of tamoxifen and its metabolite endoxifen seem to be exposure dependent, especially in younger women.^141^

While the preponderance of evidence in patients with breast cancer supports a negative cognitive impact from SERMs, it is important to note that tamoxifen has been associated with positive outcomes in postmenopausal women without cancer.^142^ This may be impacted by tamoxifen acting as a partial estrogen receptor agonist in certain tissues.^143^^, ^^144^ Fulvestrant is a selective estrogen receptor degrader, which acts by degrading the estrogen receptor and is used at time of disease progression or after tamoxifen in postmenopausal women.^133^^, ^^145^ Several studies assessing fulvestrant’s effect on cognitive outcomes showed no clearly detrimental effect, although studies often include fulvestrant in combination with the treatments, including more widely used CDK4/6 inhibitors (eg abemaciclib).^146^^, ^^147^

#### Aromatase Inhibitors

Aromatase inhibitors (eg anastrozole, letrozole) lower estrogen levels by blocking conversion from androgens.^133^ There are mixed findings with regard to the effect of aromatase inhibitors on cognition, with less evidence supporting a negative cognitive impact compared to tamoxifen.^132^ While aromatase inhibitors have been associated with cognitive impairment,^148^ multiple other clinical studies showed no difference in cognition with the use of aromatase inhibitors.^149^^, ^^150^ In a randomized clinical trial, an aromatase inhibitor (exemestane) has not shown an association with deficits in cognitive function, while patients randomized to tamoxifen did show cognitive decline in various domains, also after switching to an aromatase inhibitor.^137^^, ^^151^ The difference between cognitive outcomes with aromatase inhibitors and SERMs may be at least partly secondary to variation in mechanisms of action, in particular where SERMs may impact on tissue types based on estrogen receptor subtype and agonist versus antagonist effects.^137^^, ^^152^ Interestingly, while there was no significant cognitive decline after use of aromatase inhibitors in patients with breast cancer, Hurria et al. showed largely increased cerebral glucose metabolism on FDG PET, most robust in the mesial temporal lobe. Within this study, while hypermetabolism in occipital regions was correlated with improvements in visuospatial ability, hypermetabolism in the medial temporal region was not associated with either tested memory function or self-reported memory concerns (the latter improved over time in patients compared to controls). The authors speculate that PET hyperactivity may be compensatory and supports maintenance of cognitive function or the change in metabolic activity may not have any clinical consequence.^153^

#### Prostate Cancer

In prostate cancer, hormone therapy targets androgens (testosterone) and is utilized for advanced diseases with either metastasis or biochemical recurrence. Categories of hormone therapy for prostate cancer include androgen deprivation therapy, androgen receptor antagonists, and CYP17 inhibitors.^154^ While cognitive impairment may be more consistently reported in patients with breast cancer, and historically, constituting much of the focus for the field of CTRCI, there is a strong basis for cognitive impairment in patients with prostate cancer.^155^

#### Androgen Deprivation Therapy

Androgen deprivation therapy (ADT) with the use of LHRH agonists (eg leuprolide, goserelin) and antagonists (eg degarelix) decreases testosterone production in the testicles.^154^ Cross-sectional and large population-based studies have shown a clear link between multi domain cognitive impairment and ADT use.^66^^, ^^155–157^ However, several cohorts have showed no cognitive impact from ADT treatment.^158^ Importantly, hormone therapy for prostate cancer has also been associated with increased risk for the development of progressive cognitive decline and dementia.^159^^, ^^160^

#### Androgen Receptor Antagonists

Second-generation androgen receptor antagonists (or antiandrogens) are a newer class of hormone therapy for prostate cancer and work by blocking testosterone’s effect at androgen receptors.^161^ While there also remains debate,^162^ second-generation androgen receptor antagonists, particularly apalutamide and enzalutamide, have been associated with negative cognitive outcomes, even when compared to traditional hormone therapy.^67^^, ^^163^ A systematic review including data from 12 randomized clinical trials (>13,000 patients) found a 2-fold increased risk of cognitive toxicity from second-generation androgen receptor antagonists, as well as finding increased fatigue and fall risk.^67^ The results of the recent ODENZA trial (NCT03314324) highlight that therapy choice is important to consider, especially when there are multiple comparable options. ODENZA is a randomized phase 2 trial that assessed patient preference between 2 second-generation androgen receptor antagonists, darolutamide and enzalutamide, in men with metastatic castrate resistant prostate cancer.^164^ Although the study did not meet its primary endpoint on patient preference, the authors reported a moderate benefit in episodic memory that favored darolutamide. Likewise, there was a trend toward lower fatigue with darolutamide treatment. There is a biological rationale supporting this, as darolutamide has minimal blood-brain barrier penetration compared to enzalutamide and thus may lower the risk for direct CNS side effects of androgen receptor inhibition.^165^

#### CYP17 Inhibitors

CYP17 inhibitors (eg abiraterone) block androgen synthesis that occurs at sites other than the testicles, including adrenal glands, prostate cancer cells, and is often used with other therapies. There is limited data assessing the cognitive effects of this class of medications. However, studies have suggested that there are reduced risks of toxicity, such as fatigue and cognitive toxicity, when compared to second-generation androgen receptor antagonists.^163^^, ^^166^

### Targeted Therapies

Nearly half of all new recent FDA approvals for cancer treatment have included small-molecule drugs, with a goal of better targeting specific cancer vulnerabilities with a theoretical goal of limiting adverse effects.^167–169^ The 2 main classes of molecular targeting therapy include small-molecule agents or therapeutic monoclonal antibodies.^167^ The largest category is tyrosine kinase inhibitors, which includes EGFR inhibitors (erlotinib, osimertinib) and multi-kinase inhibitors (ibrutinib, regorafenib). Other classes of small molecule agents include proteasome inhibitors (bortezomib), cyclin-dependent kinase (CDK) inhibitors (abemaciclib, ribociclib), poly ADP-ribose polymerase (PARP) inhibitors (olaparib, niraparib), BRAF inhibitors (dabrafenib, vemurafenib), isocitrate dehydrogenase (IDH) inhibitors (ivosidenib, vorasidenib), and others, which have been previously reviewed.^167–171^ Cancer-targeted monoclonal antibodies are another class of targeted therapy.^167^ Note that the cognitive effects of a subclass of these therapeutics, immune checkpoint inhibitors, will be reviewed elsewhere. Therapeutic monoclonal antibodies work through indirect or direct mechanisms,^169^ examples include bevacizumab which binds to soluble vascular endothelial growth factor (VEGF) inhibiting binding to the VEGF receptor, human epidermal growth factor receptor 2 (HER2) targeted trastuzumab, among many other agents.^169^^, ^^172^^, ^^173^ While targeted therapies are increasingly utilized in oncology practice, less is known about their cognitive effects compared to traditional chemotherapy and hormone therapy. Additionally, this class of medications is often combined with other therapies in assessing specific contribution to cognitive outcomes can be challenging.

#### Anti-Angiogenic Therapies

Anti-angiogenic targeted therapies, namely VEGF inhibitors used in the management of multiple cancers, including glioblastoma, renal cell carcinoma, colorectal cancer,^174^ have variably been related to cognitive outcomes. In the management of glioblastoma and cerebral radiation necrosis, bevacizumab is associated with improved cognitive outcomes,^175^ related to reduction cerebral edema, mass effect, and limiting the use of higher dose co rticosteroids, which can have negative cognitive effects.^176^ Several trials of bevacizumab have shown varying results. The RTOG 0825 trial assessing bevacizumab in newly diagnosed glioblastoma showed that bevacizumab treatment was associated with worse cognitive outcomes,^177^ which differed from results of the similar AVAglio trial that showed no impact of bevacizumab on self-reported cognition,^178^ despite similar survival outcome data. Potential mechanisms that may explain cognitive worsening (particularly in normal brain) include reduction in hippocampal synaptic plasticity and neuroprotection.^179^^, ^^180^ Bevacizumab may also mask identification of tumor progression based on changes in contrast enhancement. These patients may appear radiographically stable but have disease progression and associated cognitive decline. In the management of metastatic renal cell carcinoma, anti-angiogenic therapy (sunitinib, sorafenib, bevacizumab, everolimus, pazopanib) has been associated with cognitive impairment in about 30% of patients, which may have been partly explained by increased therapy-related fatigue.^181^

#### EGFR and ALK Inhibitors

There is little known about cognitive effects of EGFR inhibitors, which are utilized primarily for the management of non-small-cell lung cancer.^182^ Kange et al. assessed cognitive outcomes in patients with non-small-cell lung cancer by treatment status (untreated, targeted therapy (EGFR inhibitors), chemotherapy). All groups had cognitive impairment in at least 1 domain (30%-35%) without a significant difference between groups.^183^ In another study, authors found that the EGFR inhibitor gefitinib did not impact cognitive symptoms (QLQ-C30).^184^ The blood-brain barrier penetration of EGFR inhibitors is variable and likely will impact CNS toxicities.^185^ ALK inhibitors are also primarily used in the management of non-small-cell lung cancer, and may variably be related to cognitive outcomes.^186^ Results from the phase 3 PROFILE 1014 trial showed that the ALK inhibitor crizotinib was associated with reduction in lung cancer symptoms, including cognition, with greater improvement in quality of life as compared to chemotherapy.^187^ Lorlatinib and alectinib are newer generation ALK inhibitors used in the treatment of ALK-positive non-small-cell lung cancer, with superior control of brain metastasis compared to crizotinib,^188^^, ^^189^ likely related to increased blood-brain barrier penetration.^190^ While lorlatinib and alectinib have provided important survival benefits, there is increased CNS toxicity, which can be seen in up to 40% of patients with lorlatinib treatment, including about 30% experiencing cognitive effects.^189^ Despite the potential for higher CNS toxicity with lorlatinib, treatment discontinuation related to adverse effects is less than with alectinib.^191^ While cognitive side effects are reported with newer generation ALK inhibitors, the fact that they lead to better CNS disease control and may delay the need for other CNS toxic treatments (eg radiation therapy), which may also impact cognitive outcomes, must be considered in clinical practice.

#### CDK4/6 Inhibitors

CDK4/6 inhibitors (palbociclib, ribociclib, abemaciclib) are increasingly utilized in the management of advanced hormone receptor-positive breast cancer and work by preventing progression of cells through the cell cycle.^192^ There currently is limited evidence assessing cognitive outcomes specifically tied to this class of medications.^132^ A recent sub study from the phase 3 SONIA trial^193^ showed greater than expected cognitive decline in a small group of patients (∼15%) during a 9 months of first-line treatment with an aromatase inhibitor, irrespective of the addition of CDK4/6 inhibitors.^194^

#### BRAF and MEK Inhibitors

There is limited evidence assessing the cognitive effects of BRAF and MEK inhibitors (alone or in combination), which is commonly used for multiple cancers with activating BRAF mutations, especially melanoma and increasingly used in primary CNS malignancies.^195^^, ^^196^ Results from the COMBI-d trial showed overall better health-related quality of life outcomes, including cognitive concerns at 40 weeks, for combination of dabrafenib and trametinib versus dabrafenib alone in patients with metastatic melanoma.^197^ The COMBI-v trial, assessing the combination of dabrafenib and trametinib versus vemurafenib alone showed similar effects on health-related outcomes, but cognitive concerns scores were worse than baseline for both groups.^198^ A recent report assessing data from the FDA adverse event reporting system in patients with colorectal cancer showed that the BRAF inhibitor encorafenib is associated with depressed mood and cognitive disorders.^199^ In the same study, the tyrosine kinase inhibitor, regorafenib, sometimes used a recurrent glioblastoma,^200^ was associated with multiple symptoms including altered state of consciousness, depressed mood, and insomnia.^199^

In the context of neurofibromatosis type 1 (NF1), use of MEK inhibitors has shown safety from neurotoxicity and cognitive outcomes are stable to improve in 48-week follow-up.^201^ These results have been supported by other studies.^202^ This is important as many of the included patients with NF1 are in childhood/adolescence, and preservation of cognition is important for continued development. A case series in pediatric patients with low-grade glioma also showed generally stable cognitive performance with MEK inhibitor (trametinib) treatment.^203^ Previous studies have shown negative cognitive and neuropsychiatric outcomes after standard management in patients with craniopharyngioma (particularly with hypothalamus involvement).^204^^, ^^205^ It will be important to monitor the cognitive outcomes of patients with BRAF V600E mutant papillary craniopharyngioma, given the recent advancement in the management with combination BRAF/MEK inhibition (vemurafenib-cobimetinib).^206–208^

#### IDH Inhibitors

Isocitrate dehydrogenase (IDH) inhibitors have been used primarily for leukemia (eg ivosidenib for AML), although given the IDH mutations present in IDH mutant gliomas (IDH mutant astrocytoma, oligodendroglioma), ivosidenib has been utilized off-label for several years in patients with glioma.^209–214^ IDH inhibitors are increasingly utilized as part of routine Neuro-Oncology practice with the recent FDA approval of vorasidenib for grade 2 IDH mutant glioma after surgery but prior to chemoradiation.^215^ Analysis from the Phase 3 INDIGO trial^216^ showed minor cognitive changes in patients who received vorasidenib, although this was similar to placebo, additionally health-related quality of life was similar between treatment arms.^217^^, ^^218^ As cognitive impairment is a prominent symptom in patients with glioma, especially in patients with low-grade glioma who have longer expected survival, it will be important to continue to monitor the cognitive effects of treatment as well as the potential benefit from delaying the need for standard chemoradiation.^21^^, ^^22^^, ^^219^

### Combination Therapies

While each individual systemic therapy may contribute to cognitive outcomes, often therapies are utilized in combination as part of a treatment regimen, or sequentially at the time of cancer recurrence or progression. This can make it challenging to attribute individual cognitive effects of a specific antineoplastic agent, which is especially important for infiltrating primary brain tumors such as glioblastoma and low-grade glioma as treatment often includes not only systemic therapy but also surgery and radiation.

## Mechanisms and Pathophysiology

**Figure 3.**
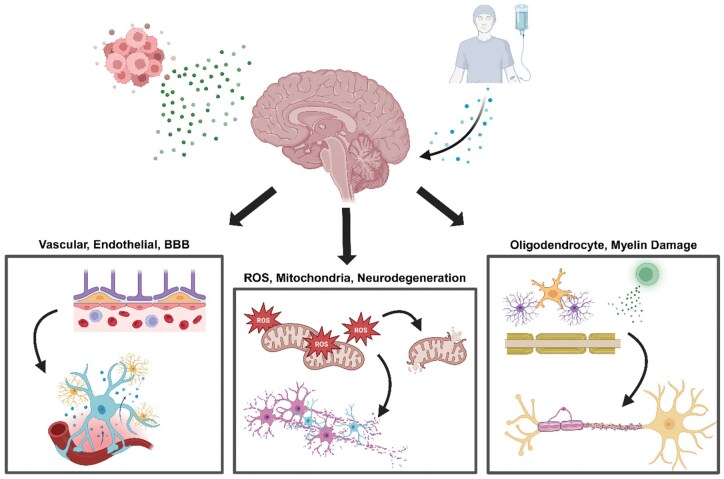
Mechanisms contributing to cancer treatment–related cognitive impairment. Multiple known mechanisms impacted by cancer treatment (and the effects of cancer itself) contribute to CTRCI. The most well recognized is systemic and neuroinflammation, which has many downstream effects, including impaired blood-brain barrier function, activation of microglia and resultant damage to neurons. Other established mechanisms include increased oxidative stress and reactive oxygen species production, mitochondrial dysfunction, and resultant neurodegeneration. Chemotherapy has also been shown to have a toxic effect on oligodendrocytes and myelination.

There has been recent progress in understanding the pathophysiologic mechanisms contributing to CRCI (Figure 3), and particularly the effects of cancer treatment on cognition-related outcomes. Similar to the multifactorial contributions to cognitive outcomes in patients with cancer, there are likely multiple pathways that contribute to CRCI.^4^^, ^^8^^, ^^16^^, ^^220^^, ^^221^ The pathways contributing to acute, subacute, and chronic cognitive sequelae related to cancer and cancer treatment may differ depending on cancer type and treatment. It is important to consider the clinical presentation, especially when determining appropriate interventions to mitigate cognitive disorders.

### Neuroinflammatory Pathways

Systemic inflammation and neuroinflammation are the most well-studied mechanisms contributing to CTRCI and the negative effects of cancer treatment.^4^^, ^^8^^, ^^221^^, ^^222^ Although systemic malignancy can promote an inflammatory milieu even prior to the start of treatment, which has been associated with worse outcomes in several cancers,^223^^, ^^224^ cancer treatments also provoke a sustained immune response, which results in elevated levels of pro-inflammatory cytokines during and after treatment.^225–227^ Multiple studies have highlighted elevation in inflammatory cytokines (eg IL-1B, IL-6, TNFa), chemokines (eg MCP-1), and cytokine receptors (eg TNFRI, TNFRII) in patients undergoing cancer treatment. Indeed, higher circulating cytokine levels have been associated with greater post treatment subjective and objective cognitive impairment.^3^^, ^^4^^, ^^8^ Williams et al. showed that higher levels of soluble TNFR1 was associated with worse short-term memory in breast cancer patients receiving chemotherapy.^228^ Janelsins et al. showed that multiple inflammatory cytokine and receptors increased over the course of chemotherapy and that increase is related to cognitive impairment. An interesting finding was that higher baseline IL-6 was associated with better post-chemotherapy cognitive performance. As this was a longitudinal study, it is possible that patients with high baseline IL-6 levels, already experiencing relative cognitive impairment that improved over time.^229^ Oppegaard et al. more comprehensively assessed inflammatory pathways (using RNAseq and microarray data) associated with self-reported cognitive concerns in patients with cancer receiving chemotherapy.^222^ They found that 5 inflammatory pathways (cytokine-cytokine receptor interaction, MAPK, IL-17, TNF, mTOR signaling pathways) were associated with self-reported attention difficulties (attentional function index, AFI).^222^ Moreover, autopsy studies have shown a long-term activation of microglial populations, oxidative stress, and DNA damage in brains of patients who have received chemotherapy.^230^^, ^^231^

Elevated systemic inflammation can impact the brain through direct and indirect mechanisms.^232^ Pro-inflammatory cytokines enter the CNS through vulnerability in the blood-brain barrier, which can be provoked by cytokine-induced breakdown of tight junctions or endothelial cell function; this increased permeability allows for circulating cytokines and even immune cells to enter the brain parenchyma.^232^^, ^^233^ Cytokines can also enter through physiological regions of the brain that lack typical brain barrier, as well as inflammatory signals transmitted through efferent nerves (eg vagus nerve).^232^^, ^^233^ Pro-inflammatory signaling leads to the activation of microglial cells, which release additional cytokines, and ultimately downstream effects, including production of reactive oxygen species (ROS)/oxidative stress, which can further activate a cycle of neuroinflammation.^232^^, ^^233^ Gibson and Monje et al. showed that methotrexate leads to the persistent activation of microglial and subsequent astrocyte activation. In a mouse model they found that microglial depletion rescues microglial dysregulation and cognitive dysfunction.^85^ Peripheral immune cells migrate into the CNS, amplifying local inflammatory response, target specific cell types, and exacerbate damage.^232^^, ^^233^ Other mechanisms contributing to neuroinflammation include disruption of the hypothalamic pituitary adrenal axis and disruption of glymphatic clearance.^232^^, ^^234–236^ Neuroinflammation also may increase synaptic dysfunction, neuronal excitability, gliosis, and neurodegeneration.^237^^, ^^238^

### Oxidative Stress and Mitochondrial Dysfunction

Chemotherapy can lead to increased production of ROS and oxidative damage to the brain.^239^ Multiple chemotherapies, including cyclophosphamide, methotrexate, carboplatin and doxorubicin, have been shown to increase oxidative stress with downstream effects including impact on mitochondrial function and direct neurotoxicity.^231^^, ^^240^^, ^^241^ Excess ROS production can lead to lipid peroxidation, protein oxygenation, and DNA strand breaks.^242^^, ^^243^ Worse cognitive outcomes have been found in older patients with multiple cancers, particularly after treatment^39–44^; this has led to hypothesis that chemotherapy may modify the normal aging trajectory (which may be related to oxidative stress/mitochondrial dysfunction) and lead to advanced brain aging.^241^^, ^^244^ Oxidative stress can promote mitochondrial dysfunction through damage of mtDNA another processes. However, antineoplastic agents, including traditional chemotherapy and targeted therapy, directly affect mitochondrial function by promoting negative downstream sequelae.^245^^, ^^246^

### Impaired Neurogenesis and Neuroplasticity

As traditional chemotherapeutic agents affect DNA, chemotherapy also directly damages DNA in normal brain cells, particularly vulnerable are proliferating neural progenitor cells that can trigger apoptotic cell death.^231^^, ^^247^ Multiple chemotherapeutic agents, including cyclophosphamide, 5-FU, cisplatin, have been shown to disrupt hippocampal neurogenesis.^248^^, ^^249^ This affects the brain’s capacity to form new neurons and maintain synaptic connections and may contribute to the reduction of cognitive reserve/resilience and the ability to withstand future injury.^250^ In mouse models, cisplatin and doxorubicin lead to reduced dendritic branching and loss of dendritic spines, which is associated with impaired learning and memory. These effects were shown to be related to BDNF signaling, which could be augmented by riluzole.^251^^, ^^252^

### Vascular and Endothelial Injury

Antineoplastic therapies have long been known to have vascular effects with cardiovascular toxicities, including hypertension, cardiomyopathy, heart failure, arrhythmias, amongst others.^253^ This also includes indirect effects on the cerebral vasculature from thromboembolism as well as direct toxic effects on endothelial cells that can impact normal cerebral perfusion and blood-brain barrier function.^253^^, ^^254^ Cisplatin specifically has been associated with endothelial damage, thromboembolic events, and promoting endothelial senescence.^255^ Long-term endothelial dysfunction and microvascular damage may create chronic hypoxic environment in the brain that can exacerbate neuronal stress and cognitive symptoms.^256^^, ^^257^

### Hormonal and Neuroendocrine Influences

Hormone therapy for the management of breast and prostate cancer has important cognitive implications.^66^^, ^^132^ While there remains debate impact of anti-estrogen hormone therapy, estrogens clearly have a key role in normal brain function and disruption of normal levels or downstream effects may lead to adverse effects.^258^^, ^^259^ Estrogen has been shown to modulate neural differentiation, regulation of brain networks, and synaptic plasticity.^260–263^ Additionally, estrogen has a neuroprotective effect that contributes to resilience against age-related cognitive decline (eg Alzheimer’s disease), with evidence supporting improvement in regulating neuroinflammation and mitochondrial function.^264^^, ^^265^ An important distinction with the use of hormone therapy in breast cancer is timing of administration. Premenopausal women have higher levels of circulating estrogen and the use of anti-estrogen therapy in this population may be more likely to worsen cognition,^266^ although specific studies assessing pre and postmenopausal breast cancer survivors are limited. A recent study examined the relationship between cognitive outcomes, tamoxifen dosage, and plasma levels of tamoxifen and its active metabolite, endoxifen, across several cognitive domains.^141^ In that study, older women were particularly susceptible to tamoxifen-related cognitive impairment. These results align with TEAM trial data,^137^ which also revealed tamoxifen-related deficits in verbal memory and executive function among older women. Interestingly, the exposure-response association was more pronounced in younger women, while it was largely absent in older participants. This may reflect an age-related ceiling effect: older women may experience reduced cognitive function at relatively low exposure levels, while younger women may remain stable at low doses but decline at higher exposure levels.

Similar to estrogen, testosterone has a demonstrated role in brain health and cognitive function through various functions, including support of neurogenesis, synaptic plasticity, regulation of neurotransmitter function, reduced oxidative stress, and improved mitochondrial function.^267–269^ Androgen receptors are found throughout the brain, most commonly in the hippocampus, amygdala, and prefrontal cortex, further supporting that testosterone plays an important role in brain function.^269^^, ^^270^ The pathophysiologic mechanisms contributing to cognitive symptoms and impairment in patients receiving hormone therapy in prostate cancer are most likely multifactorial. Indeed, hormone therapy exacerbates multiple other symptoms that may impact cognitive function, including fatigue, mood changes, and metabolic and cardiovascular dysfunction.^271–275^

Cancer treatments can also impact physiologic endocrine function through direct impact on the hypothalamic-pituitary adrenal (HPA) axis.^276^ Chemotherapy may also affect normal hormone production in other organs, including ovarian suppression in premenopausal women, which may contribute to cognitive symptoms.^277^^, ^^278^

## Biomarkers

While at present there are no established biomarkers for CTRCI, given the increased understanding of potential pathophysiologic mechanisms, multiple candidate biomarkers provide insight into which patients are at risk for cognitive impairment. It is likely that biomarkers will differ based on cancer type and treatment exposure. Below, we summarize objective measures that have been related to cognitive outcomes in cancer survivors.

### Neuroimaging

Neuroimaging is an important candidate biomarker for the assessment of CTRCI.^279^ Studies of structural MRI have shown a reduction in gray matter volume (particularly frontal and temporal) and density in cancer survivors after treatment, which has been associated with cognitive outcomes.^280–284^ Research using diffusion tensor imaging has shown decreased fractional anisotropy and increased mean diffusivity after chemotherapy, suggesting that disruption in white matter microstructural integrity may represent a possible neuroimaging biomarker of CTRCI.^285–287^ Studies assessing functional MRI (fMRI) have shown disrupted functional connectivity, particularly with the disruption of the default mode network in cancer survivors.^288–294^

FDG PET is widely used for evaluation of dementia and can provide important diagnostic information with regard to underlying etiology of cognitive symptoms/impairment.^295^^, ^^296^ However, there are limited data using FDG PET assessing cerebral metabolism in patients with cancer, despite the fact that many patients undergo FDG PET imaging as part of routine clinical practice.^297^^, ^^298^ Several studies have shown cerebral hypometabolism after exposure to antineoplastic agents.^153^^, ^^299–302^ Given the utility of FDG PET for assessing multiple cognitive etiologies, future use of this modality may provide further insights into CTRCI.

### Biofluid Markers

There is significant interest in relating blood and cerebrospinal fluid-based measures to cognitive outcomes.^303^ There is a significant literature showing that elevated inflammatory markers (most commonly IL6, TNFα, soluble TNF receptors, IL1β, CRP) relate to worse cognitive outcomes in cancer survivors, thus this is an area of interest.^304^^, ^^305^ Recent studies have also related Alzheimer’s disease blood and CSF biomarkers to cognitive outcomes in cancer survivors, with mixed findings, particularly given limited data assessing these measures.^306^^, ^^307^

### Genetic Assessment

Several genetic polymorphisms have been related to increased risk for the development of cognitive impairment in cancer survivors. Carriage of APOE4 is a well-established risk factor for the development of Alzheimer’s disease.^48^^, ^^49^ Likewise, APOE4 has been shown in multiple studies to be related to worse cognitive outcomes in patients with cancer, and after specific cancer-targeted treatment exposures, including chemotherapy^39^^, ^^50–53^ and radiation.^54^ While APOE genotype is not routinely assessed to determine risk of outcomes in cancer survivors, as a case example, APOE genotype is now routinely assessed in the clinical assessment of patients with Alzheimer’s disease as the risk for complications (amyloid-related imaging abnormality) from treatment with new anti-amyloid therapies (eg lecanemab, donanemab) is impacted by APOE genotype (eg APOE4 homozygous patients have increased risk for therapy-related complications).^308^ Obtaining APOE genotype should be a clinical consideration in Oncology practice, particularly when clinicians are using therapies that are associated with an increased risk for cognitive sequelae, and/or with future clinical trial enrollment (eg identifying higher risk patients). Several other genetic polymorphisms, including MTHFR, GNB3, COMT, and BDNF, have also been previously associated with cognitive outcomes in cancer survivors.^55–58^^, ^^157^^, ^^304^^, ^^309^ Consideration of genetic testing for established genetic polymorphisms increasing risk for CTRCI and treatment-related effects may one day provide better information for informed treatment decision-making and potential clinical trial enrollment.

## Challenges and Future Directions

We remain with limited preventative strategies and supportive therapeutic options once patients develop symptoms, despite considerable progress in understanding pathophysiologic contributors to CTRCI and the effects of cancer treatments.^8^^, ^^16^ We know that common risk factors for neurodegenerative disorders (eg Alzheimer’s) also appear to be risk factors for CTRCI (eg age, APOE4). However, we have a limited understanding of individual patient-level risk factors for the development of cognitive impairment after antineoplastic treatment. Several key reasons for this include: (1) heterogenous nature of CTRCI—many cancer types, varied disease stage and treatments; (2) lack of established biomarkers, which not only limits clinical diagnosis but also the assessment of therapeutic response of established and trial therapies; (3) inadequately accounting for the multifactorial contributors to subjective cognitive concerns and objective cognitive impairment in cancer survivors, both clinically and in research settings. By focusing on these gaps, we have the opportunity to develop a more refined understanding of CTRCI and the effects of cancer treatments, which will help guide clinical decision-making and potential future therapeutic options.

Other important limitations regarding our current understanding of cognitive outcomes in cancer patients include: (1) lack of longitudinal cognitive assessment at baseline, throughout treatment, and post-treatment; (2) variability in how cognition is assessed in clinical practice and research studies (eg self-report vs. neuropsychologic testing; variable test and questionnaire selection); (3) heterogeneity of cohorts (eg including multiple cancer types, different disease stages, and treatment exposures); (4) some objective tests may not be sensitive enough to detect objective impairment in patients with cognitive symptoms; (5) lack of standardized cognitive endpoints. Harmonization of cognitive evaluation guided by consensus recommendations (ICCTF, RANO-COG) and guidelines (NCCN) can provide a more robust way to compare cognitive outcomes between studies of specific cancer populations and treatment exposures. There is also variability and sometimes discordance between subjective cognitive concerns and objective cognitive impairment.^3^^, ^^4^^, ^^16^^, ^^20^^, ^^30^^, ^^31^ While both impact quality of life and functional status,^5^^, ^^9^^, ^^26–29^ patients often report significant cognitive symptom and can be in the “normal range” on objective testing. Subjective and objective measures of cognitive function are complementary. Evaluation that only incorporates either subjective or objective measures may not adequately represent the level of cognitive burden that patients manage. Thus, it is imperative for future studies to include both subjective and objective measures, particularly in studies with prospective longitudinal assessment and clinical trials.

Improved understanding of CRCI and the cognitive effects of cancer treatment provides patients and their clinicians with better insight into the potential toxicities of cancer treatment, as well as supportive interventions and future trial opportunities that may improve cognitive impairment. The goal of any intervention aimed at ameliorating the effects of cancer treatment should not negatively affect the benefit gained from a specific therapy but rather support improved cognitive functioning and quality of life.

## Conclusion

Cognitive impairment is an important concern for cancer survivors, reflecting the reality that the goal of cancer treatment is not only to improve the quantity but also to improve the quality of life. While the potential contributors to CRCI are often multifactorial, among the most clinically relevant is treatment-related cognitive impairment, as this can have implications for cancer management and long-term outcomes. With increased basic and clinical research into the pathophysiologic mechanisms contributing to CTRCI, the historical view of chemobrain or chemo fog is becoming a more well-defined entity. Future research should focus on understanding the subjective and objective longitudinal cognitive trajectories, accounting for individual patient-level and clinical/treatment factors, harmonizing routine cognitive assessments, and working on establishing biomarkers that may help guide future targeted preventative and supportive therapeutic trials aimed at improving cognitive outcomes and quality of life in cancer survivors.

## Author Contributions

Bryan Neth, Sanne Schagen, and Jeffrey Wefel (Manuscript conception, Writing—review and approval)

## Conflict of Interest Statement

Bryan Neth declares no potential conflicts of interest. Sanne Schagen declares a leadership role in the Dutch Neuropsychological Society. Jeffrey Wefel declares grants from MDACC and NCI and consulting fees from Astellas, Bayer, Intracellular Therapeutics, GCAR, GT Medical Technology, Novocure.
